# Structure–Activity Relationship of New Chimeric Analogs of Mastoparan from the Wasp Venom *Paravespula lewisii*

**DOI:** 10.3390/ijms23158269

**Published:** 2022-07-27

**Authors:** Jarosław Ruczyński, Brygida Parfianowicz, Piotr Mucha, Katarzyna Wiśniewska, Lidia Piechowicz, Piotr Rekowski

**Affiliations:** 1 Laboratory of Chemistry of Biologically Active Compounds, Faculty of Chemistry, University of Gdańsk, Wita Stwosza 63, 80-308 Gdańsk, Poland; b.parfianowicz@gmail.com (B.P.); piotr.mucha@ug.edu.pl (P.M.); piotr.rekowski@ug.edu.pl (P.R.); 2 Department of Medical Microbiology, Faculty of Medicine, Medical University of Gdańsk, Dębowa 25, 80-204 Gdańsk, Poland; katarzyna.wisniewska@gumed.edu.pl (K.W.); lidia.piechowicz@gumed.edu.pl (L.P.)

**Keywords:** antimicrobial studies, circular dichroism studies, antimicrobial peptides, mastoparan, transportan, RNAIII-inhibiting peptide

## Abstract

Mastoparan (MP) is an antimicrobial cationic tetradecapeptide with the primary structure INLKALAALAKKIL-*NH_2_*. This amphiphilic α-helical peptide was originally isolated from the venom of the wasp *Paravespula lewisii*. MP shows a variety of biological activities, such as inhibition of the growth of Gram-positive and Gram-negative bacteria, as well as hemolytic activity and activation of mast cell degranulation. Although MP appears to be toxic, studies have shown that its analogs have a potential therapeutic application as antimicrobial, antiviral and antitumor agents. In the present study we have designed and synthesized several new chimeric mastoparan analogs composed of MP and other biologically active peptides such as galanin, RNA III inhibiting peptide (RIP) or carrying benzimidazole derivatives attached to the ε-amino side group of Lys residue. Next, we compared their antimicrobial activity against three reference bacterial strains and conformational changes induced by membrane-mimic environments using circular dichroism (CD) spectroscopy. A comparative analysis of the relationship between the activity of peptides and the structure, as well as the calculated physicochemical parameters was also carried out. As a result of our structure–activity study, we have found two analogs of MP, MP-RIP and RIP-MP, with interesting properties. These two analogs exhibited a relatively high antibacterial activity against *S. aureus* compared to the other MP analogs, making them a potentially attractive target for further studies. Moreover, a comparative analysis of the relationship between peptide activity and structure, as well as the calculated physicochemical parameters, may provide information that may be useful in the design of new MP analogs.

## 1. Introduction

The development of drug resistance is one of the biggest problems of antimicrobial chemotherapy which results, among other things, from excessive usage of antimicrobial agents. Nowadays, the number of resistant bacteria to traditional antibiotics is growing extremely rapidly, which makes the treatment of serious infections such as sepsis or meningitis very difficult. Antibiotic-resistant microorganisms are a leading cause of infections that result in high morbidity and death rates. Overcoming the problem of resistance during the treatment of serious infections would, beyond any doubt, have a positive impact on the final outcome of these therapies. Thus, the development of new compounds with improved antimicrobial activities against drug-resistant microorganisms is one of the major challenges in medicinal chemistry.

In recent years, many research groups have focused their attention on antimicrobial peptides (AMPs) as a novel class of antibiotics [1,2,3,4,5,6,7,8,9]. These peptides, as an important element of the body’s natural immunity against microorganisms, are widespread in nature and exhibit a broad spectrum of antimicrobial activity and low susceptibility to the development of drug resistance [1,2,3]. Generally, AMPs are relatively short cationic amphipathic peptides consisting of 5–40 amino acids in their sequence, which carry a net positive charge (usually 2–10 charged residues) [1,2,3,4,5,6,7,8,9]. Their amino acid sequences are composed of hydrophilic and hydrophobic residues, whereby they are soluble in both the aqueous and lipid phases [5,7]. In water, AMPs are largely unstructured, but tend to adopt α-helical or β-sheet secondary structures upon contact with the hydrophilic/hydrophobic membrane of microorganisms [7,8].

AMPs kill pathogens through lethal cell permeabilization or inhibition of intracellular targets (e.g., inhibition of protein or nucleic acid biosynthesis, protease activity) [1,2,3,4,5,6,7,8,9]. The antimicrobial activity of AMPs seems most likely to be a consequence of their interaction with the bacterial membrane, including translocation across the membrane, association into clusters, or the formation of pores on the membrane (including the carpet model, barrel stave model, toroidal pore model or “detergent”-like model) [1,2,5,6,7,8,9]. The exact mechanism of action of antimicrobial peptides against pathogens is unknown. However, it is well known that the physicochemical properties of peptides, such as length, net charge, charge distribution, hydrophobicity, amphipathicity, conformation and oligomeric state in solution, play important roles in the interactions of peptides with the microbial membrane [3,5,6,7].

One of the most promising groups of AMPs are peptides belonging to the mastoparan family [10,11,12,13,14,15,16,17,18,19,20,21,22,23,24,25,26]. Mastoparan (MP) is amphiphilic α-helical tetradecapeptide with an amidated *C*-terminus (INLKALAALAKKIL-*NH_2_*), originally isolated from the venom of the wasp *Paravespula lewisii* [10,11,19,20,22,24]. Some MP analogs (tetradecapeptides with different amino acids sequences) have also been isolated from the venom of hornets, yellow jackets, and paper or solitary wasps [11,12,13,17,20]. They all are rich in hydrophobic amino acids such as Leu, Ile, and Ala, and commonly possess Lys residues. MP shows a variety of biological activities such as the inhibition of the growth of Gram-positive and Gram-negative bacteria, activation of mast cell degradation and histamine release, activation of phospholipase A_2_ and C, and erythrocyte lysis. MP also enhances the permeability of artificial and biological membranes and activates GTP-binding regulatory proteins by a mechanism analogous to that of G protein-coupled receptors [10,12,17,18,20,21,22,24,25]. In addition, several studies have demonstrated the antitumor and antiviral activity of MP and its analogs in vitro [10,13,21]. Although mastoparan appears to be toxic, studies have shown that its analogs have a potential therapeutic applications as antimicrobial, antiviral and antitumor agents (presumably because of their toxicity) [11,21]. MP as a component of cell-penetrating peptides (CPPs) has a potential pharmaceutical application in the delivery of different molecules (such as plasmids, DNA, siRNA, PNA, proteins, peptides and low-molecular-weight drugs) into cells without damaging them [18,21,22,27,28,29,30].

Due to such obstacles as low stability or half-life in human serum, toxicity of AMPs against eukaryotic cells, as well as potential immunogenicity (i.a., high hemolytic activity and promoting massive mast cell degranulation), only a few of these peptides have been approved as antimicrobial agents in clinics [2,6,7,8,9,10,14,25]. Therefore, several strategies to reduce the toxic side effects of AMPs and improve their antimicrobial activities have been proposed, including modification of peptide charge, alteration of the sequence length, replacement of amino acids with D-isomers or unnatural structures, modulation of peptide hydrophobicity, modification of *N*-and *C*-terminus, or alteration of the α–helical structure content [2,3,5,9,10,15,17,21,25]. So far, many mastoparan analogs have been synthesized, and although some of them contain a mastoparan sequence in their primary structure, they have lost some of the properties of MP [10,11,14,15,18,19,22,23,24,25,26].

On the other hand, some studies using designed synthetic antimicrobial peptides, especially short and ultra-short AMPs, have shown their great antimicrobial potential against various strains of bacteria and fungi, including drug-resistant strains. Among them, very interesting properties have been shown by cathelicidin analogs with a truncated sequence and containing D-amino acid substitutions [31]. One of them, 19-amino acid HC1-D2 analog, retained potent, broad-spectrum and rapid antimicrobial properties against bacteria and fungi, especially drug-resistant bacteria. Moreover, this peptide exhibited high stability in human serum, antibiofilm and anti-inflammatory activity, low propensity to induce bacterial resistance and low cytotoxicity and hemolytic activity. It also displayed potent in vivo anti-infective ability in a mouse peritonitis model infected with both standard and drug-resistant bacteria. In other studies, Sharma et al. demonstrated the potent antifungal activity of synthetic ultra-short peptides, amphipathic peptides arylated at histidine residues [32]. The most potent analog with the sequence His(2-biphenyl)-Trp-His(2-biphenyl) showed high activity against *C. neoformans*, high proteolytic stability and low hemolytic activity as well as a synergistic effect in combination with amphotericin B and fluconazole. However, studies with the use of temporin L analogs proved that the stabilization of the α-helical conformation by introducing various intramolecular bridges (such as lactam, disubstituted [1,2,3]-triazole, hydrocarbon or disulfide) leads to an increase in the helical fraction content and thus improves antimicrobial activity. Among the large number of synthesized analogs, one analog of temporin L, containing a lactam bridge formed between the Lys^6^ and Glu^10^ residues, showed particularly interesting properties. This analog was characterized by potent antimicrobial activity against a wide spectrum of bacterial strains, including the drug-resistant strain of *S. aureus*. Moreover, it showed high antibiofilm activity, low cytotoxicity and high stability in human serum [33].

**Figure 1:**
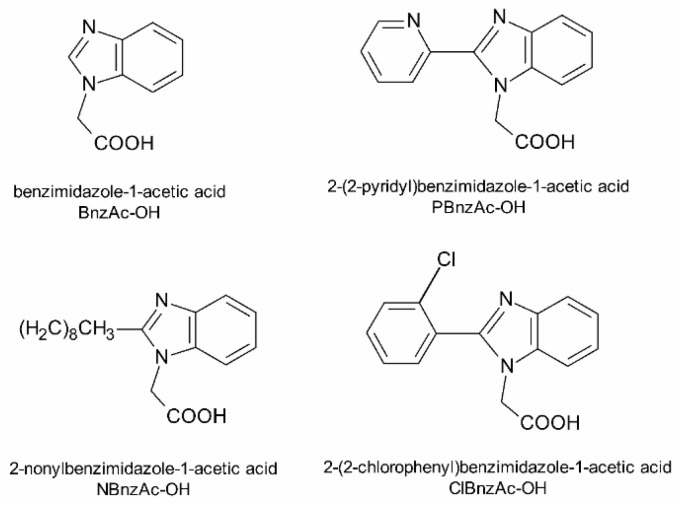
Structures of synthesized benzimidazole derivatives attached to TP10 sequence.

In this study we have designed and synthesized several new chimeric mastoparan analogs composed of MP and other biologically active peptides (galanin, RNA III inhibiting peptide) or carrying benzimidazole derivatives attached to the ε-amino side group of Lys residue (Figure 1 and Table 1). Next, we examined each of these hybrid constructs as well as natural peptides for their antimicrobial activities against three reference bacterial strains: *Staphylococcus aureus*, *Escherichia coli* and *Pseudomonas aeruginosae*. We also applied circular dichroism (CD) spectroscopy to evaluate the conformational changes of the peptides induced by membrane-mimic environments. We expected that such modifications would improve the peptides’ ability to inhibit bacterial proliferation, and this may lead to finding new therapeutic agents.

## 2. Results

### 2.1. Design and Synthesis of Peptides

In the present studies we have designed and synthesized several new chimeric MP analogs composed of MP and other biologically active peptides, their analogs or carrying unnatural structures (Table 1). All peptides were synthesized by solid phase peptide synthesis with the use of the standard Fmoc strategy. Thus, we obtained an MP analog with a reversed amino acid sequence (retroMP) and its chimera consisting of a native MP sequence in the *N*-terminal part and a retroMP sequence in the *C*-terminal part. It is believed that retro peptides may retain the biological activity of native peptides while increasing their resistance to proteolysis [22]. The other peptides designed were the chimeric MP analogs composed of MP and RNA III inhibiting peptide (RIP) or its more effective analog ([Lys^2^, Ile^4^] RIP) linked to the *N*-or *C*-terminus of the MP sequence. Although RIP and its analogs do not kill bacteria directly, they are known to reduce toxin synthesis (by inhibition of RNA III synthesis) and cell adhesion in vitro, and may be effective inhibitors of infections caused by all strains of *S. aureus* [34,35,36,37,38]. Furthermore, to investigate the effect of the deletion of amino acid residues on the antimicrobial properties of MP, we have also synthesized an analog of MP with a truncated sequence at the *N*-terminus (by removing the first three residues) and its chimeric analogs with a RIP or [Lys^2^, Ile^4^] RIP attached to its *C*-terminus.

Another group of chimeric MP analogs include peptides obtained by coupling a galanin (Gal) fragment to the *N*-terminus of the mastoparan (e.g., Gal(1–13)-MP—known as galparan or Galp). Among them, the chimeric analogs composed of the *N*-terminal fragment of galanin 1–12 and mastoparan at the *C*-terminus linked with Lys residue (transportan, TP) or its truncated analog (transportan 10, TP10) are of particular interest. TP and TP10 are well known as cell-penetrating peptides (CPPs) and have great potential due to their ability to penetrate the cell membrane and deliver different molecules into cells [27,28,29,30]. In addition, TP10 has much lower cytotoxicity than MP and exhibits significant antimicrobial activity alone as well as part of a conjugate with other molecules, such as vancomycin, for example [28]. Taking this into account, we designed and synthesized a series of new TP10 conjugates with RIP or [Lys^2^, Ile^4^] RIP attached to its *C*-terminus or benzimidazole derivatives (Figure 1) attached via a methylenecarbonyl linker to the ε-amino side chain group of Lys^7^ residue. Benzimidazoles are of great interest due to their broad spectrum of biological activity, including antimicrobial, antiviral and anticancer properties [39,40,41,42,43,44].

The physicochemical properties (such as molecular weight, number of residues, net charge, content of hydrophobic residues, mean hydrophobicity, mean hydrophobic moment and instability index) of the synthesized peptides are presented in Table 2. The mean hydrophobicity (H) of each peptide was calculated as the average of hydrophobicities of each amino acid in the peptide sequence using the Fauchere and Pliska hydrophobicity scale [45]. A positive H value suggests that the peptide has some hydrophobicity. However, the mean hydrophobic moment (*µ*H), used as a quantitative measure of amphipathicity, was calculated according to the Eisenberg formula [46,47] using the normalized Eisenberg consensus hydrophobicity scale [48]. A large value *µ*H means that the peptide has amphipathic properties [17]. Peptide instability indexes were predicted by ProtParam in ExPASy http://web.expasy.org/protparam/ (accessed on 1 June 2022). An instability index of less than 40 indicates that the peptide is stable.

### 2.2. Antimicrobial Activity Study

A comparison of the MIC values (minimal inhibitory concentration) for the tested peptides, reflecting their antimicrobial potency against the three reference bacterial strains, is presented in Table 3. Our results confirmed the relatively strong antimicrobial properties of MP and its chimeric TP10 analog, as well as the low antimicrobial activity of two other known MP chimeric analogs, Galp and TP. Relatively low concentrations of mastoparan inhibit the growth of *Staphylococcus aureus* (8 µg/mL) and *Escherichia coli* (32 µg/mL). Moreover, after transferring the broth culture onto the solid medium, no growth of *S. aureus* and *E. coli* was observed. Thus, the MP concentrations used in the study were simultaneously bactericidal. Additionally, TP10 had a relatively low concentration (16 µg/mL) that inhibited the growth of *S. aureus*. In the case of the other two bacterial strains, the effect of TP10 was significantly weaker.

Lack of antimicrobial activity (up to a peptide concentration of 256 µg/mL) was shown by analogs in which the mastoparan sequence was truncated by removing the first three *N*-terminal amino acid residues (MP (4–14) fragment). Similarly, peptides containing the retro sequence (retroMP analog and its MP-retroMP chimera) did not show activity against the tested bacterial strains up to a concentration of 256 µg/mL. Contrary to our expectations, none of the TP10 analogs containing benzimidazole derivatives in the lysine side chain at position 7 displayed antimicrobial activity (in the tested concentration range) against the bacterial strains used. In line with previous literature reports and our expectations, RIP and its [Lys^2^, Ile^4^] RIP analog were inactive against the tested strains. However, the combination of RIP and [Lys^2^, Ile^4^] RIP with mastoparan and transportan 10 resulted in the formation of chimeric peptides with interesting antibacterial properties.

All analogs obtained by combining MP or TP10 with RIP or [Lys^2^, Ile^4^] RIP showed the highest antimicrobial activity against *S. aureus*. The peptides with attached RIP to the *N*- or *C*-terminus of mastoparan had the highest antibacterial activity against *S. aureus*. The MP-RIP analog turned out to be the most active in microbiological tests, among all the peptides tested. The value of the lowest concentration inhibiting the growth of *S. aureus* for this analog was 16 µg/mL and was somewhat higher than that for MP, and similar to that for TP10 (or comparable to that for MP or TP10 when expressed as molar concentration). However, unlike MP and TP10, MP-RIP was practically not active against the other two bacterial strains. The situation was different in the case of the RIP-MP analog—the MIC value against *S. aureus* was 32 µg/mL and was twice as high as that for MP-RIP. In contrast, the MIC value for *E. coli* was 128 µg/mL, which was half that for MP-RIP.

The peptides obtained by attaching [Lys^2^, Ile^4^] RIP to the *C*- or *N*-terminus of the mastoparan have similar antibacterial activity against *S. aureus* (MIC = 64 µg/mL). Their activity was much weaker than that observed in the case of MP-RIP and RIP-MP. However, MP-[Lys^2^, Ile^4^] RIP had the highest MIC value among MP analogs against the tested *E. coli* strain. TP10-RIP and TP10-[Lys^2^, Ile^4^] RIP also showed higher activity against the *S. aureus* strain, but their antimicrobial activity was significantly weaker than that observed for TP10. The concentration values that inhibit microbial growth for these peptides were significantly higher than those for TP10 and were 128 µg/mL. Removal of the Lys residue at position 7 from the amino acid sequence of the peptide consisting of TP10 and [Lys^2^, Ile^4^] RIP, thereby obtaining the [desLys^7^] TP10-[Lys^2^, Ile^4^] RIP analog, increased the antimicrobial activity of the peptide compared to the parent analog TP10-[Lys^2^, Ile^4^] RIP. The MIC value against the *S. aureus* strain decreased two-fold to 64 µg/mL.

### 2.3. Circular Dichroism Study

**Figure 2:**
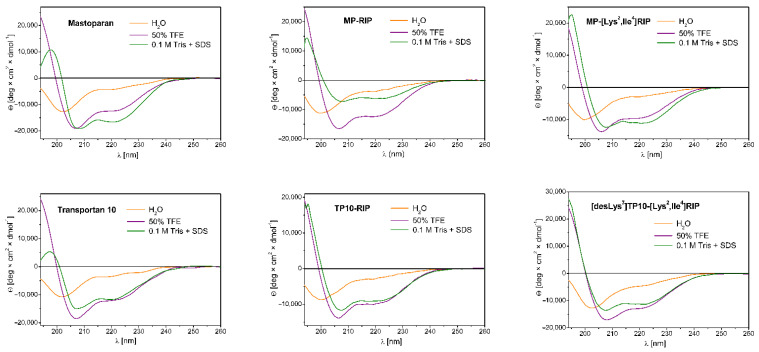
Examples of CD spectra in water (orange line), 50% TFE (purple line) and 0.1 M Tris buffer with the addition of 50 mM SDS (olive line) for MP and its selected analogs.

Circular dichroism spectroscopy was applied to evaluate the conformational changes of peptides induced by membrane-mimetic environments. The secondary structures of MP and some of its MP analogs (Table 4) were determined in water, 50% 2,2,2-trifluoroethanol (TFE) and 0.1 M Tris buffer with the addition of 50 mM SDS. The relative α-helix fraction (*f*_H_) values presented in Table 4, calculated from the values of mean residue ellipticity at 222 nm, reflect the ability of peptides to adopt helical structure in the tested environments. Examples of CD spectra for selected MP analogs are shown in Figure 2.

The spectra of MP and its analogs in water showed a negative band near 200 nm, indicating an unordered conformation of the peptides corresponding to the dominant random coil structure. For such analogs as MP-retroMP, MP (4–14), MP (4–14)-RIP and MP (4–14)-[Lys^2^, Ile^4^] RIP, we observed a significant decrease in the intensity of this band with a simultaneous shift in the direction of shorter wavelengths. The *f*_H_ values for these peptides ranged from 0.02–0.04. In contrast, the highest helical content induced in water was observed for retroMP, RIP-MP, [Lys^7^ (PBnzAc)] TP10 and MP (*f*_H_ = 0.14–0.19).

In the 50% TFE environment, we observed significant changes in the CD spectra of all peptides tested. The spectra were characterized by the presence of two negative bands (deeper at about 208 nm and shallower at about 222 nm) and a distinct positive band at about 193 nm, indicating the tendency of peptides to adopt helical structures in a membrane—mimetic environment. The lowest tendency to adopt a helical structure was demonstrated for three analogs: MP-retroMP, MP (4–14)-RIP, MP (4–14)-[Lys^2^, Ile^4^] RIP—the *f*_H_ values for these peptides were in the range 0.23–0.24. On the other hand, the highest tendency to adopt the helical structure was shown by peptides such as: [Lys^7^ (PBnzAc)] TP10 (*f*_H_ = 0.51), as well as RIP-MP, unmodified MP, [Lys^2^, Ile^4^] RIP-MP, MP-RIP, Galp, TP, TP10, and [desLys^7^] TP10-[Lys^2^, Ile^4^] RIP (*f*_H_ = 0.34–0.38).

In the 0.1 M Tris environment with the addition of 50 mM SDS, we observed a diverse tendency of the peptides to form helical structures. The presence of SDS micelles is intended to simulate the lipid membrane environment and to favor the tendency of peptides to adopt an ordered structure. As in the case of the 50% TFE environment, the CD spectra in SDS were also characterized by two negative bands (one near 208 nm and the other near 222 nm) and a maximum near 193 nm. Overall, the peptides showed a lower or similar tendency to adopt helical structures compared to that in 50% TFE, with some exceptions. The highest tendency (higher than that in 50% TFE) was shown by MP and its truncated analog MP (4–14), with *f*_H_ values of 0.50 and 0.64, respectively. On the other hand, a surprisingly low (lower than that in water) tendency was shown by the two chimeric analogs Galp and TP, with *f*_H_ values of 0.09 and 0.10, respectively.

**Figure 3:**
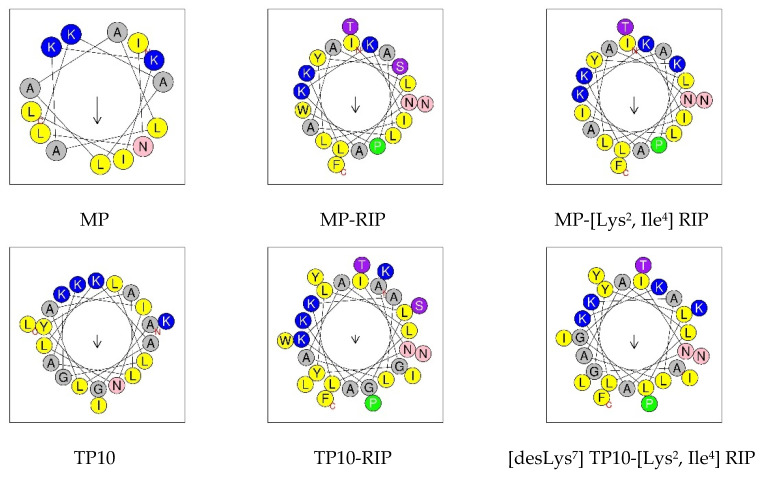
Examples of helical wheel projection for MP and its selected analogs. In this view through the helix axis, the hydrophilic residues (K, N, G, S, T) are located on one side and the hydrophobic residues (L, A, I, F, W, Y) on the other side of the helix. The arrow shows the hydrophobic moment (*µ*H) vector. N—*N*-terminus; C—*C*-terminus. Helical wheel projections were made with HeliQuest https://heliquest.ipmc.cnrs.fr/ (accessed on 1 June 2022).

Figure 3 shows a comparison of the predicted helical wheel projections for the MP and its selected analogs. Such models support the hypothesis that helix formation enables peptides to present hydrophilic and hydrophobic faces. In this view through the helix axis, the hydrophilic residues (K, N, G, S, T) are located on one side and the hydrophobic residues (L, A, I, F, W, Y) on the other side of the helix. The arrow shows the hydrophobic moment (*µ*H) vector, the value of which is a quantitative measure of amphipathicity.

### 2.4. Cytotoxicity Study

**Figure 4:**
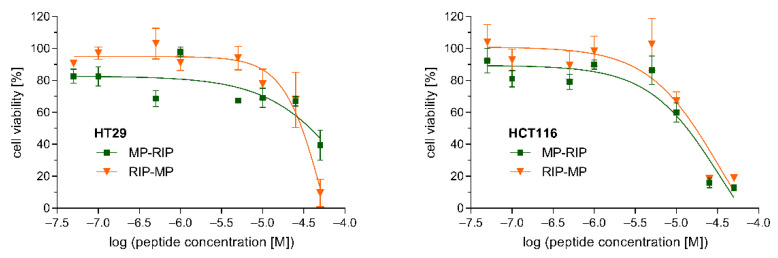
Effects of MP-RIP and RIP-MP on the viability of HT29 (**left**) and HCT116 (**right**) cells. Cells were incubated with various concentrations of peptides for 24 h and cell viability was assessed by MTT assay. Plots present mean ± SEM from three independent experiments performed in triplicate. *X* axis represents peptide concentration in logarithmic scale. *Y* axis represents cell viability expressed as a percentage relative to the untreated control cells incubated without peptides.

The cytotoxicity of the selected MP analogs, MP-RIP and RIP-MP, was determined against two human cell lines. The viability of HT29 and HCT116 cells incubated with various concentrations of MP-RIP and RIP-MP was assessed by the MTT assay (Figure 4). Both cell lines showed similar sensitivity to the tested peptides after 24 h of incubation. Our studies demonstrated that peptides up to a concentration of 10 µM did not substantially affect the viability of these cells (Figure 4). Moreover, based on the regression curves of cell viability, lethal doses of MP analogs sufficient to kill 50% of the peptide-treated cells (LD_50_) were calculated. In the case of HT29 cells, MP-RIP showed slightly lower cytotoxicity compared to the RIP-MP analogue (LD_50_ values of 38.0 µM and 30.2 µM, respectively). In contrast, the cytotoxicity of MP-RIP against HCT116 cells was slightly higher than that of RIP-MP (LD_50_ values of 13.2 µM and 17.0 µM, respectively). The viability of the HT29 cells indicates relatively low cytotoxicity of both analogs at a concentration corresponding to the MIC value against *S. aureus*. In the case of HCT116 cells, the above relationships were less evident.

## 3. Discussion

When designing new antimicrobial peptides, the physicochemical properties of peptides, such as amino acid sequence and peptide length, net positive charge and its distribution, hydrophobicity, amphipathicity, and the content of α-helical structure in a membrane-mimic environment, should be taken into account [9,17,25]. Structure–activity studies have shown that truncation of the peptide sequence at the *N*- or *C*-terminus results in a gradual decrease in antimicrobial potential. On the other hand, elongation of the peptide sequence, *N*-acylation or *C*-amidation, as well as substitutions with various unnatural amino acids, may reduce the susceptibility of the peptides to proteolytic degradation and increase their half-life in human serum. The net positive charge has been considered essential for the activity of AMPs, although the number of positive charges required for activity is position-dependent [25]. Some studies have reported that a decrease in the net charge to a level lower than +4 renders the peptide inactive, but a systematic increase in this property from +4 to +8 can make the peptide exhibit hemolytic activity.

Recent studies suggest that one of the most important physicochemical parameters may be the hydrophobic moment (*µ*H), as a quantitative measure of amphipathicity [17,25]. It is suggested that increasing the hydrophobic moment causes a significant increase in the permeabilizing and hemolytic activity of peptides [17]. However, mean hydrophobicity (H) is a measure of the affinity of a peptide to the interior of the membrane needed for effective membrane permeabilization [17]. Studies have suggested that hydrophobicity at very low levels abolishes antimicrobial activity, at high levels enhances hemolysis and at too-high levels causes aggregation and precipitation of peptides. Moreover, it is well known that in the presence of various membrane-mimic environments, the α-helical fraction content may vary depending on the hydrophobicity of the peptide (in an electrically neutral medium, e.g., TFE) or the net charge of the peptide (in an anionic micellar environment, e.g., SDS) [9,17,25]. Overall, the content of the α-helical structure affects the interaction of peptides with negatively charged membranes and, as suggested, there is a good correlation between the helical content and the antimicrobial activity of peptides.

In the present studies, we designed and synthesized several mastoparan analogs (Table 1) and then compared their antimicrobial activity and conformational changes induced by membrane-mimic environments (in TFE and SDS). Based on the obtained results, we analyzed the relationship between the antimicrobial activity of peptides (Table 3) and their physicochemical properties (Table 2) as well as the α-helical fraction content in various environments (Table 4).

The conducted studies confirmed the potent antibacterial properties of MP against *S. aureus* and *E. coli*. With regard to *P. aeruginosae*, both MP and its analogs were inactive in the range of the tested concentrations. The relatively higher activity of MP against Gram-positive compared to Gram-negative bacteria probably results from differences in their membrane structure. The antimicrobial activity of MP appears to be well correlated with its physicochemical properties such as optimal net positive charge (+4), relatively high hydrophobicity and amphipathicity. Studies with the use of circular dichroism showed a low tendency of this peptide to adopt the α-helical conformation in water and a relatively high tendency in the electrically neutral environment of 50% TFE, and even higher in the environment of anionic SDS micelles. This suggests that in the case of MP, electrostatic interactions may be mainly responsible for the stabilization of the α-helix and the interaction with the negatively charged bacterial membrane (e.g., Gram-positive *S. aureus*), causing its perturbation and damage. On the other hand, the relatively high hydrophobicity of MP is probably responsible for the stabilization of α-helix in the TFE environment and suggests that the hydrophobic interactions may be more important for interaction with the zwitterionic membrane of eukaryotic cells, causing a toxic effect, e.g., hemolytic activity [17,25]. The helical wheel projection confirmed the preferred location of hydrophobic amino acids located on one side of the helix and on the other side of hydrophilic amino acids.

On the other hand, the retroMP analog and its hybrid MP-retroMP as well as MP (4–14) and its hybrids with RIP or [Lys^2^, Ile^4^] RIP did not exhibit antibacterial activity against the tested strains in the range of the concentrations used. They also showed a similar, but lower than MP, tendency to adopt the α-helical conformation in both 50% TFE and SDS (lower in SDS than in the TFE environment, except the MP (4–14) analog). These observations suggest that the alteration of sequence length or the increase in net positive charge is not important to maintain or enhance the antimicrobial activity of MP and to stabilize the α-helical structure, but rather the distribution of both positively charged and hydrophobic amino acids in the primary structure may be essential.

Among the chimeric peptides obtained by attaching RIP or [Lys^2^, Ile^4^] RIP to the *N*- or *C*-terminus of MP, the MP-RIP analog showed the highest antibacterial activity against *S. aureus* (the MIC value comparable to that for MP when expressed as molar concentration), followed by RIP-MP and both MP hybrids with [Lys^2^, IIe^4^] RIP. In the case of the *E. coli* strain, the above-mentioned peptides were also active but showed lower antimicrobial activity in the following order of decreasing potency: MP-[Lys^2^, Ile^4^] RIP > RIP-MP > MP-RIP ≈ [Lys^2^, Ile^4^] RIP-MP. In contrast, RIP and [Lys^2^, Ile^4^] RIP were inactive against the tested bacterial strains in the concentration range used. The chimeric MP analogs, which had the highest microbial activity, also exhibited a relatively high tendency to adopt the α-helical structure in the electrically neutral environment of TFE and in the environment of anionic SDS micelles (generally lower than in TFE).

Moreover, cytotoxicity study showed relatively low toxicity of MP-RIP and RIP-MP at concentrations corresponding to their antimicrobial concentrations against *S. aureus*. The LD_50_ values for these two analogs, in particular for HT29 cells, were higher than that of TP10 (demonstrated in our previous studies) [30]. However, comparing their cytotoxic properties with other MP analogs known from the previous literature reports appears to be difficult due to the different cell lines used in cytotoxicity assays. In many cases, an increase in antimicrobial activity also resulted in an increase in cytotoxicity. For example, one of the MP analogs widely known as mitoparan (MitP), with high antimicrobial activity against different bacterial strains [18], also showed significantly increased cytotoxicity against eukaryotic cells [18,22,24]. On the other hand, MitP analogs with reduced cytotoxicity exhibited low antimicrobial activity [18]. The retroMP analog, known for its 3.5-fold lower cytotoxicity than MP [22], was completely inactive in our antimicrobial studies.

Comparing the antimicrobial properties of the above-mentioned analogs with physicochemical parameters, it seems that there is a good correlation between the activity of these analogs and their hydrophobicity (relatively high for MP-RIP and RIP-MP) and the stabilization of the α-helical structure in a hydrophobic environment. This suggests that the hydrophobicity and hydrophobic interactions may be more important than the net positive charge or amphipathicity for the interaction of these analogs with the bacterial membrane and their antimicrobial activity. Increasing the net charge from +4 to +5 as well as alteration of the hydrophobic moment seem to be of little importance. It is worth noting that attaching another peptide sequence to the *N*-terminus of the MP significantly reduces the latter parameter.

Our studies confirmed very weak antimicrobial activity of galparan (Galp) and transportan (TP) against *S. aureus*, and relatively high activity of TP10 against *S. aureus* and lower against *E. coli*. The CD spectra showed a high tendency of these analogs to adopt an α-helical structure in the TFE environment and a low tendency in the SDS environment (with the exception of TP10). TP10 was also characterized by a significantly increased hydrophobic moment and slightly reduced hydrophobicity. The removal of the first six amino acid residues in the TP sequence, which in turn increased TP10 amphipathicity and stabilized the α-helical structure in the SDS environment, probably improved its antimicrobial activity. On the other hand, the attachment of RIP or its analog [Lys^2^, Ile^4^] RIP to TP10 caused a significant decrease in the antibacterial activity of TP10 against *S. aureus* and completely abolished its activity against *E. coli*. Surprisingly, the [desLys^7^] TP10-[Lys^2^, Ile^4^] RIP analog, obtained by removing the Lys residue at position 7, which is crucial for the penetrating properties of TP10, showed two-fold greater activity against *S. aureus* than the parent analog. Among the abovementioned TP10 analogs, the [desLys^7^] TP10-[Lys^2^, Ile^4^] RIP analog was also characterized by higher hydrophobicity and hydrophobic moment, as well as the content of the α-helical fraction in the environment of 50% TFE. However, complete inactivity of TP10 analogs with attached benzimidazole derivatives turned out to be disappointing. In the case of benzimidazole derivatives, their presence in the TP10 structure probably caused large structural changes, thus disrupting the interaction of the analogs with the bacterial membrane and causing their inactivity.

In conclusion, despite the promising antimicrobial properties of MP, its use in clinical therapy is limited due to its toxicity. Therefore, there is still a need to find new MP analogs with improved properties, i.e., high and selective antimicrobial activity and low toxicity towards mammalian cells. In recent years, many analogs of MP have been synthesized, but so far little is known about chimeric analogs of MP isolated from *Paravespula lewisii* wasp venom. As a result of our preliminary structure–activity studies of new chimeric MP analogs, we found two peptides, MP-RIP and RIP-MP, with interesting properties. These peptides showed a relatively high antimicrobial activity against *S. aureus* compared to the other MP analogs, making them a potentially attractive target for further studies (the MIC value for MP-RIP was comparable to that of MP). Both analogs also showed low cytotoxicity at antimicrobial concentrations. Due to the presence in their structure of the RIP sequence capable of inhibiting the synthesis of toxins and cell adhesion in all staphylococcal strains, they may be promising compounds that would be effective in the treatment of infections caused by drug-resistant *S. aureus* strains. However, further studies are needed covering their antimicrobial efficacy against drug-resistant *S. aureus* strains, toxicity to eukaryotic cells, and stability in human serum. Based on the circular dichroism study, we showed that MP-RIP and RIP-MP (as well as TP10) are characterized by a relatively high content of the α-helical fraction (comparable to that of MP) in the electrically neutral hydrophobic environment of TFE. We believe that our comparative analysis of the relationship between peptide activity and structure, as well as the calculated physicochemical parameters, may provide information that may be useful in the design of new MP analogs.

## 4. Materials and Methods

### 4.1. Reagents

All reagents and solvents were of analytical or HPLC grade (Merck KGaA, Darmstadt, Germany). Solutions were freshly prepared with distilled deionized water using a Milli-Q Millipore system (Bedford, MA, USA) and filtered with a 0.22 μm filter before use. Fmoc (fluorenyl-9-methoxycarbonyl) protected L-amino acids used for peptide synthesis were obtained from Bachem AG (Bublendorf, Switzerland). Rink-Amide TentaGel S RAM resin was purchased from Rapp Polymere GmbH (Tuebingen, Germany).

### 4.2. Preparation of Benzimidazole Derivatives

Benzimidazole derivatives (BnzAc-OH, NBnzAc-OH, PBnzAc-OH and ClBnzAc-OH) were obtained by reacting *t*-butyl bromoacetate (1 eq) with an appropriate substrate (1.5 eq): benzimidazole, 2-nonylbenzimidazole, 2-(2-pyridyl) benzimidazole and 2-(2-chlorophenyl) benzimidazole in the presence of K_2_CO_3_ (1.2 eq) and catalytic amounts of tetrabutylammonium iodide (TBAI). Reactions were carried out in acetonitrile (ACN) for 20 h at 60 °C. After the syntheses had been completed, *t*-butyl groups were removed using 50% trifluoroacetic acid (TFA) in dichloromethane (DCM) for 2 h at room temperature. Crude products were purified by column chromatography on silica gel using methanol:ethyl acetate as the eluent. Fractions of the highest purity (>98%) were analyzed by an analytical reversed-phase high-performance liquid chromatography (RP-HPLC) system (Prominence, Shimadzu, Duisburg, Germany) using a Kinetex XB-C18 column (Phenomenex, 4.6 × 150 mm, 5 µm particle size) with several gradients of ACN with 0.08% TFA at a flow rate of 1 mL/min. Identities of compounds were confirmed by MALDI-TOF mass spectrometry (Bruker BIFLEX III, Bruker Daltonics, Billerica, MA, USA) using the α-CCA (α-cyano-4-hydroxycinnamic acid) matrix. Thus, the obtained benzimidazole derivatives (Figure 1) were attached on-resin to the ε-amino group of Lys^7^ residue within the TP10 structure.

### 4.3. Peptide Synthesis and Purification

All peptides (Table 1) were synthesized in an automated peptide synthesizer (Quartet, Protein Technologies, Tucson, AZ, USA) by applying Fmoc chemistry. TentaGel S RAM resin (loading 0.25 mM/g) for peptide amides was used as the starting material. Fmoc-protected amino acids were assembled as active derivatives in a 3-fold molar excess of *O*-(benzotriazole-1-yl)-1, 1, 3, 3-tetramethyluronium tetrafluoroborate (TBTU) with the addition of *N*-hydroxybenzotriazole (HOBt) and *N*-methylmorpholine (NMM) (1:1:2) in the *N*,*N*-dimethylformoamide (DMF) solution for 2 × 30 min. Removal of the Fmoc group was carried out with 20% piperidine-DMF in 2 cycles (2 × 3.5 min). In the case of TP10 analogs carrying benzimidazole derivatives in the side chain, a hydrazine-labile 1-[4, 4-dimethyl–2, 6-dioxocyclohex-1-ylidene)-3-methylbutyl (*iv*Dde) group was used to protect the ε-amino function group at Lys^7^ residue instead of the standard acid-labile *tert*-butyloxycarbonyl (Boc) group. As 2% hydrazine monohydrate/DMF removes the Fmoc group, the *N*-terminal of Ala residue was coupled as Boc-protected amino acid. After removal of the *iv*Dde group, all benzimidazole derivatives were attached to the ε-amino group of Lys^7^ using the same method as for peptide chain assembly. The synthesized peptides were cleaved from the peptidyl resin by treating them with a mixture of TFA/phenol/water/triisopropylsilane (88/5/5/2) at room temperature for 2 h under argon bubbles. After lyophilization, the crude peptides were purified by a preparative RP-HPLC system (SpotPrep, Armen, Brittany, France) using a Reprosil 100 C-18 column (Dr. Maisch GmbH, 40 × 250 mm, 10 µm particle size). Several gradients of ACN with 0.08% TFA, at a flow rate of 25 mL/min, were used for purification. Fractions of the highest purity (>95%) were analyzed by an analytical RP-HPLC system (Prominence, Shimadzu, Tokyo, Japan) using a Kinetex XB-C18 column (Phenomenex, 150 × 4.6 mm, 5 µm particle size) with several gradients of ACN with 0.08% TFA at a flow rate of 1 mL/min (Figures S1–S4). Identities of compounds were confirmed by MALDI-TOF mass spectrometry (Bruker BIFLEX III, Bruker Daltonics, Billerica, MA, USA) using the α-CCA matrix (Figures S1–S4 and Table 4).

### 4.4. Antimicrobial Assay

Antimicrobial activity of peptides was examined against three reference strains: *Staphylococcus aureus* NCTC 4163, *Escherichia coli* NCTC 8196 and *Pseudomonas aeruginosae* NCTC 6749 dedicated for the determination of antibacterial activity. The bacterial strains were stored in glycerol stock (Sigma-Aldrich, St. Louis, MO, USA) at −80 °C. Before testing, the strains were cultured aerobically in Tryptic Soy Agar (BD DIFCO, Franklin Lakes, NJ, USA) at 37 °C for 20 h. The susceptibility of microorganisms to the peptides was determined by the broth microdilution assay according to the procedures outlined by CLSI [49]. The final concentration of the peptides prepared in wells with Mueller–Hinton broth ranged from 0.125 to 256 µg/mL. In order to prepare the bacterial suspension, an overnight culture of bacteria in Tryptic Soy Broth (BD DIFCO, Franklin Lakes, NJ, USA) was diluted in sterile saline to a final concentration of approximately 10^7^ CFU/mL of bacteria. Aliquots (5 µL) of bacterial suspension were added to each peptide solution. The sterility control (containing the tested peptide in broth) and the growth control (containing the reference strain in broth without the tested peptide) were also set up. The plates were incubated at 37 °C under aerobic conditions. The results are expressed as the minimal inhibitory concentration (MIC) of the compounds in question. The MIC was defined as the lowest concentration at which no visible growth of bacteria (no turbidity) was observed. The assay was performed in triplicate.

### 4.5. Circular Dichroism Spectroscopy

Circular dichroism (CD) spectra of peptides were recorded on a Jasco J-815 spectropolarimeter (Jasco Int. Co., Ltd., Tokyo, Japan). The CD spectra were acquired for peptides in a concentration of 0.15 mg/mL in water, 50% 2,2,2-trifluoroethanol (TFE) or 0.1 M tris(hydroxymethyl)aminomethane (Tris) buffer (pH 7.01) with the addition of 50 mM SDS (sodium dodecylsulphate) in 0.1 cm path length cuvettes. Measurements were taken at 25 °C in a spectral range of 193–260 nm with a bandwidth of 1 nm and response of 1 s. The spectra were averaged after three scans. Ellipticity was measured in mdeg units and converted to mean residue ellipticity (Θ). The relative helix content (*f*_H_) was calculated from the mean residue ellipticity values at 222 nm using the method proposed by Rohl and Baldwin [50] and described previously [51]. Helical wheel projections (Figure 3) were made with HeliQuest https://heliquest.ipmc.cnrs.fr/ (accessed on 1 June 2022).

### 4.6. Cytotoxicity Assay

Human cell lines HT29 (ATCC cat. # HTB-38) and HCT116 (ATCC cat. # CCL-247) were cultured in McCoy (HT29) or Dulbecco’s Modified Eagle’s (DMEM) medium (HCT116), supplemented with 10% fetal bovine serum (FBS), penicillin (100 U/mL) and streptomycin (100 µg/mL). Cultures were conducted at 37 °C in humidified atmosphere of 5% CO_2_. Cells were cultured for 24 h before initiation of the experiments. The cell viability was determined by the 3-(4, 5-dimethylthazol-2-yl)-2, 5-diphenyl tetrazolium bromide (MTT) assay. Briefly, HT29 or HCT116 cells were seeded in 96-well plates to a density of 10^5^ cells/well in 0.2 mL of above-specified culture media. After 24 h, the following concentrations of peptides were added to wells: 0.05, 0.1, 0.5, 1, 5, 10, 25, 50 µM. As a negative control, cells were treated with 0.9% NaCl. After 24 h of incubation (5% CO_2_, 37 °C), 15 *µ*L MTT solution was added into each well to reach the final concentration of 10 mg/mL. After further incubation for 2 h (5% CO_2_, 37 °C) the cells were centrifuged for 3 min at 4 °C and the medium was removed. Formazan crystals present in mitochondria of metabolically active cells were dissolved with 100 *μ*L of dimethylsulfoxide (DMSO), and the dye concentration was determined at 570 nm wavelength. Cell viability was expressed as a percentage (mean value ± SEM) of those cells treated with 0.9% NaCl as negative control. Lethal doses, which correspond to peptide concentrations sufficient to kill 50% of the peptide-treated cells (LD_50_), were determined with GraphPad Prism 7.0 software (GraphPad Software Inc., La Jolla, CA, USA). The assay was performed in triplicate.

## Figures and Tables

**Table 1:** Primary structures of synthesized peptides.

Peptide	Amino Acid Sequence
MP	INLKALAALAKKIL-*NH_2_*
retroMP	LIKKALAALAKLNI-*NH_2_*
MP-retroMP	INLKALAALAKKILLIKKALAALAKLNI-*NH_2_*
RIP	YSPWTNF-*NH_2_*
MP-RIP	INLKALAALAKKILYSPWTNF-*NH_2_*
RIP-MP	YSPWTNFINLKALAALAKKIL-*NH_2_*
MP (4–14)	KALAALAKKIL-*NH_2_*
MP (4–14)-RIP	KALAALAKKILYSPWTNF-*NH_2_*
[Lys^2^, Ile^4^] RIP	YKPITNF-*NH_2_*
MP (4–14)-[Lys^2^, Ile^4^] RIP	KALAALAKKILYKPITNF-*NH_2_*
MP-[Lys^2^, Ile^4^] RIP	INLKALAALAKKILYKPITNF-*NH_2_*
[Lys^2^, Ile^4^] RIP-MP	YKPITNFINLKALAALAKKIL-*NH_2_*
Gal (1–13)-MP (Galp)	GWTLNSAGYLLGPINLKALAALAKKIL-*NH_2_*
Gal (1–12)-Lys-MP (TP)	GWTLNSAGYLLGKINLKALAALAKKIL-*NH_2_*
Gal (7–12)-Lys-MP (TP10)	AGYLLGKINLKALAALAKKIL-*NH_2_*
TP10-RIP	AGYLLGKINLKALAALAKKILYSPWTNF-*NH_2_*
TP10-[Lys^2^, Ile^4^] RIP	AGYLLGKINLKALAALAKKILYKPITNF-*NH_2_*
[desLys^7^] TP10-[Lys^2^, Ile^4^] RIP	AGYLLGINLKALAALAKKILYKPITNF-*NH_2_*
[Lys^7^ (BnzAc)] TP10	AGYLLGK (BnzAc) INLKALAALAKKIL-*NH_2_*
[Lys^7^ (NBnzAc)] TP10	AGYLLGK (NBnzAc) INLKALAALAKKIL-*NH_2_*
[Lys^7^ (PBnzAc)] TP10	AGYLLGK (PBnzAc) INLKALAALAKKIL-*NH_2_*
[Lys^7^ (ClBnzAc)] TP10	AGYLLGK (ClBnzAc) INLKALAALAKKIL-*NH_2_*

BnzAc—benzimidazole-1-acetyl; ClBnzAc—2-(2-chlorophenyl) benzimidazole-1-acetyl; Gal—galanin; Galp—galparan; MP—mastoparan; NBnzAc—2-nonylbenzimidazole-1-acetyl; PBnzAc—2-(2-pyridyl) benzimidazole-1-acetyl; RIP—RNA III-inhibiting peptide; TP—transpoprtan; TP10—transportan 10.

**Table 2:** The physicochemical properties of synthesized peptides.

Peptide	MW	N	Q	^%^H	H	*µ*H	Ins
MP	1478.91	14	+4	71.43	0.576	0.398	10.91
retroMP	1478.91	14	+4	71.43	0.576	0.398	28.54
MP-retroMP	2940.81	28	+7	71.43	0.576	0.241	20.09
RIP	912.98	7	+1	57.14	0.763	nd	2.81
MP-RIP	2374.88	21	+4	66.67	0.639	0.292	8.69
RIP-MP	2374.88	21	+4	66.67	0.639	0.093	8.69
MP (4–14)	1138.49	11	+4	72.73	0.470	0.598	18.88
MP (4–14)-RIP	2034.46	18	+4	66.67	0.584	0.359	13.19
[Lys^2^, Ile^4^] RIP	881.03	7	+2	57.14	0.563	nd	−45.14
MP (4–14)-[Lys^2^, Ile^4^] RIP	2002.50	18	+5	66.67	0.506	0.379	−5.46
MP-[Lys^2^, Ile^4^] RIP	2342.92	21	+5	66.67	0.572	0.312	−7.30
[Lys^2^, Ile^4^] RIP-MP	2342.92	21	+5	66.67	0.572	0.090	−7.30
Galp	2809.42	27	+4	62.96	0.631	0.134	6.33
TP	2840.48	27	+5	59.26	0.567	0.173	0.04
TP10	2181.76	21	+5	66.67	0.560	0.235	−1.52
TP10-RIP	3077.73	28	+5	64.29	0.610	0.152	−0.08
TP10-[Lys^2^, Ile^4^] RIP	3045.78	28	+6	64.29	0.560	0.165	−12.07
[desLys^7^] TP10-[Lys^2^, Ile^4^] RIP	2917.60	27	+5	66.67	0.618	0.249	−9.74
[Lys^7^ (BnzAc)] TP10	2339.76	21	+4	nd	nd	nd	nd
[Lys^7^ (NBnzAc)] TP10	2466.76	21	+4	nd	nd	nd	nd
[Lys^7^ (PBnzAc)] TP10	2417.36	21	+4	nd	nd	nd	nd
[Lys^7^ (ClBnzAc)] TP10	2450.36	21	+4	nd	nd	nd	nd

MW—calculated molecular weight (g/mol); N—number of residues; Q—net charge at pH 7.4; ^%^H—content of hydrophobic residues (%); H—mean hydrophobicity; *µ*H—mean hydrophobic moment; Ins—instability index; nd—not determined.

**Table 3:** The antimicrobial activity of synthesized peptides against three reference bacterial strains.

Peptide	MIC				
	*S. aureus*		*E. coli*		*P. aeruginosae*
	[μg/mL]	[μM]	[μg/mL]	[μM]	[μg/mL]
MP	8	5.4	32	21.6	256
retroMP	>256	>172.8	256	172.8	256
MP-retroMP	>256	>87.5	>256	87.5	>256
RIP	>256	>280.4	>256	>280.4	>256
MP-RIP	16	6.7	256	107.2	>256
RIP-MP	32	13.4	128	53.6	>256
MP (4–14)	>256	>224.4	>256	>224.4	>256
MP (4–14)-RIP	>256	>125.8	>256	>125.8	>256
[Lys^2^, Ile^4^] RIP	>256	>290.6	>256	>290.6	>256
MP (4–14)-[Lys^2^, Ile^4^] RIP	256	127.8	>256	>127.8	>256
MP-[Lys^2^, Ile^4^] RIP	64	27.8	64	27.8	>256
[Lys^2^, Ile^4^] RIP-MP	64	27.8	256	109.3	>256
Galp	128	45.5	>256	>91.1	>256
TP	128	45.1	>256	>90.1	>256
TP10	16	7.3	128	58.7	256
TP10-RIP	128	41.6	>256	>83.2	>256
TP10-[Lys^2^, Ile^4^] RIP	128	42.0	>256	>84,0	>256
[desLys^7^] TP10-[Lys^2^, Ile^4^] RIP	64	21.9	256	87.6	>256
[Lys^7^ (BnzAc)] TP10	256	109.4	>256	>109.4	>256
[Lys^7^ (NBnzAc)] TP10	>256	>103.8	>256	>103.8	>256
[Lys^7^ (PBnzAc)] TP10	>256	>105.3	>256	>105.3	>256
[Lys^7^ (ClBnzAc)] TP10	>256	>104.5	>256	>104.5	>256
Gentamicin	0.5	1.1	1.0	2.1	0.25

MIC—minimal inhibitory concentration; gentamicin—standard drug control.

**Table 4:** The relative α-helix fraction (*f*_H_) calculated from the mean residue ellipticity values at 222 nm for the selected peptides.

Peptide	Relative α-Helix Fraction (*f*_H_)		
	Water	50% TFE	0.1 M Tris + 50 mM SDS
MP	0.14	0.38	0.50
retroMP	0.19	0,27	0.16
MP-retroMP	0.04	0.24	0.17
MP-RIP	0.11	0.34	0.18
RIP-MP	0.15	0.39	0.23
MP (4–14)	0.02	0.31	0.64
MP (4–14)-RIP	0.05	0.23	0.23
MP (4–14)-[Lys^2^, Ile^4^] RIP	0.04	0.23	0.18
MP-[Lys^2^, Ile^4^] RIP	0.09	0.27	0.32
[Lys^2^, Ile^4^] RIP-MP	0.10	0.35	0.31
Galp	0.13	0.34	0.09
TP	0.12	0.34	0.10
TP10	0.10	0.34	0.32
TP10-RIP	0.08	0.27	0.25
TP10-[Lys^2^, Ile^4^] RIP	0.08	0.29	0.31
[desLys^7^] TP10-[Lys^2^, Ile^4^] RIP	0.13	0.34	0.25
[Lys^7^ (PBnzAc)] TP10	0.15	0.51	0.32

SDS—sodium dodecylsulfate; TFE—2,2,2-trifluoroethanol; Tris—tris(hydroxymethyl)aminomethane.

## Data Availability

The datasets used and/or analyzed during the current study are available from the corresponding author on reasonable request.

## Supplementary Materials

The following supporting information can be downloaded at: https://www.mdpi.com/article/10.3390/ijms23158269/s1.

## Abbreviations

AMPs	antimicrobial peptides
BnzAc	benzimidazole-1-acetyl
CD	circular dichroism
ClBnzAc	2-(2-chlorophenyl) benzimidazole-1-acetyl
CPP	cell-penetrating peptide
FBS	fetal bovine serum
*f*_H_	relative α-helix fraction
Gal	galanin
Galp	galparan
H	mean hydrophobicity
HPLC	high-performance liquid chromatography
LD_50_	peptide concentration sufficient to kill 50% of the peptide-treated cells
*μ*H	mean hydrophobic moment
MIC	minimal inhibitory concentration
MitP	mitoparan
MP	mastoparan
MTT	3-(4, 5-dimethylthazol-2-yl)-2, 5-diphenyl tetrazolium bromide
NBnzAc	2-nonylbenzimidazole-1-acetyl
PBnzAc	2-(2-pyridyl) benzimidazole-1-acetyl
RIP	RNA III inhibiting peptide
SDS	sodium dodecylsulfate
TFE	2,2,2-trifluoroethanol
TP	transportan
TP10	transportan 10
Tris	tris (hydroxymethyl) aminomethane
