# Supplementary material for: Structure–Activity Relationship of New Chimeric Analogs of Mastoparan from the Wasp Venom Paravespula lewisii
Source: Int J Mol Sci. 2022 Jul 27;23(15):8269. doi: 10.3390/ijms23158269 (PMC9332802; doi:10.3390/ijms23158269)
Supplement: Supplementary file 1 [file ijms-23-08269-s001.zip › ijms-1815628-supplementary.pdf]

Structure-Activity Relationship of New Chimeric Analogs of Mastoparan From the Wasp Venom *Paravespula Lewisii*

Jarosław Ruczyński [1,*], Brygida Parfianowicz [1], Piotr Mucha [1], Katarzyna Wiśniewska [2], Lidia Piechowicz [2] and Piotr Rekowski [1]

[1] Laboratory of Chemistry of Biologically Active Compounds, Faculty of Chemistry, University of Gdańsk, Wita Stwosza 63, 80-308 Gdańsk, Poland; piotr.rekowski@ug.edu.pl
[2] Department of Medical Microbiology, Faculty of Medicine, Medical University of Gdańsk, Dębowa 25, 80-204 Gdańsk, Poland; lidia.piechowicz@gumed.edu.pl
* Correspondence: jaroslaw.ruczynski@ug.edu.pl; Tel.: +48-58-5235431

Supporting information

Figure S1. HPLC chromatogram (up) and MALDI-TOF mass spectrum (down) of mastoparan.
Figure S2. HPLC chromatogram (up) and MALDI-TOF mass spectrum (down) of MP-RIP.
Figure S3. HPLC chromatogram (up) and MALDI-TOF mass spectrum (down) of RIP-MP.
Figure S4. HPLC chromatogram (up) and MALDI-TOF mass spectrum (down) of TP10.
Table S1. Calculated and found molecular weight of synthesized peptides.

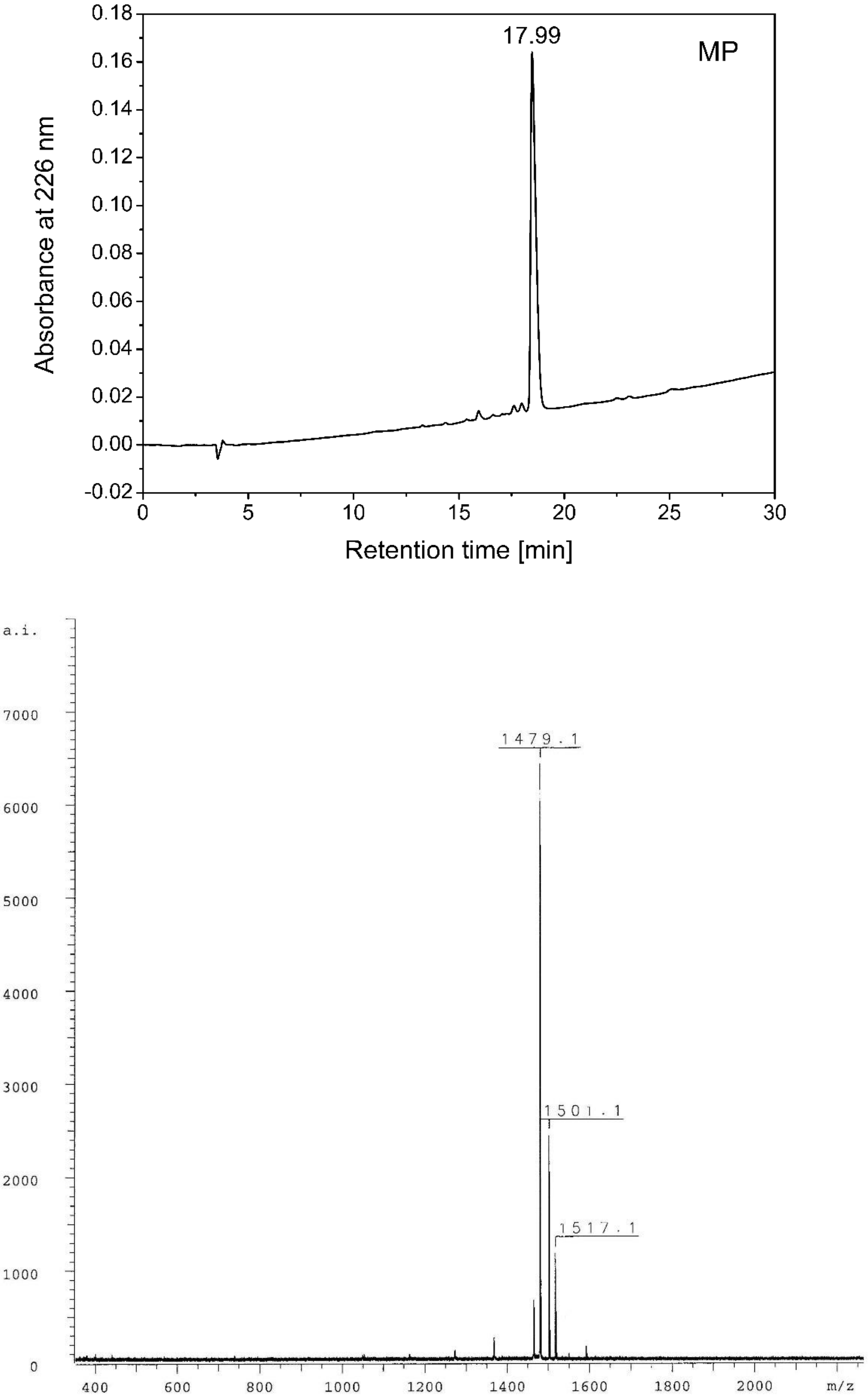

Figure S1. HPLC chromatogram (up) and MALDI-TOF mass spectrum (down) of mastoparan.

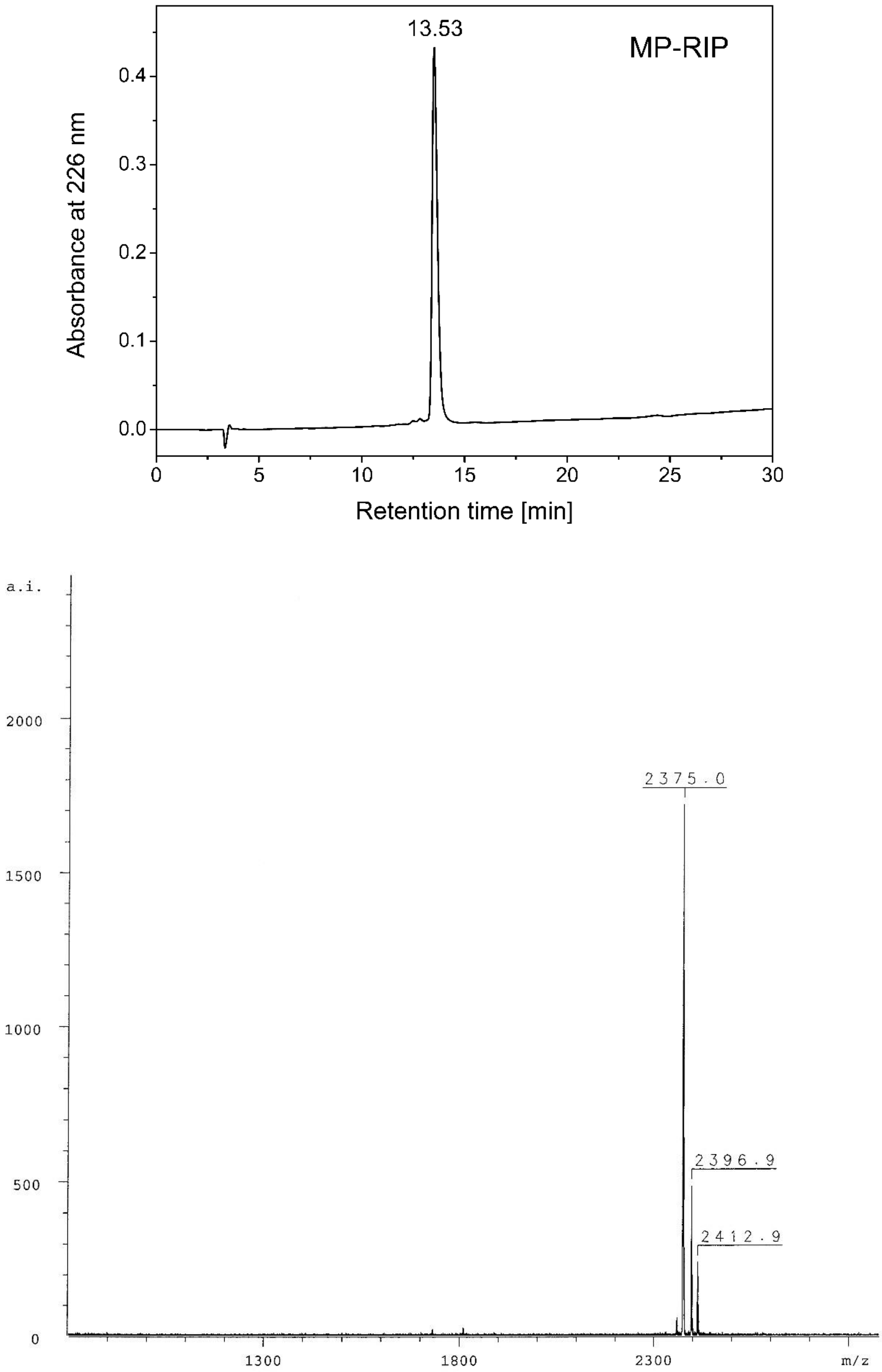

Figure S2. HPLC chromatogram (up) and MALDI-TOF mass spectrum (down) of MP-RIP.

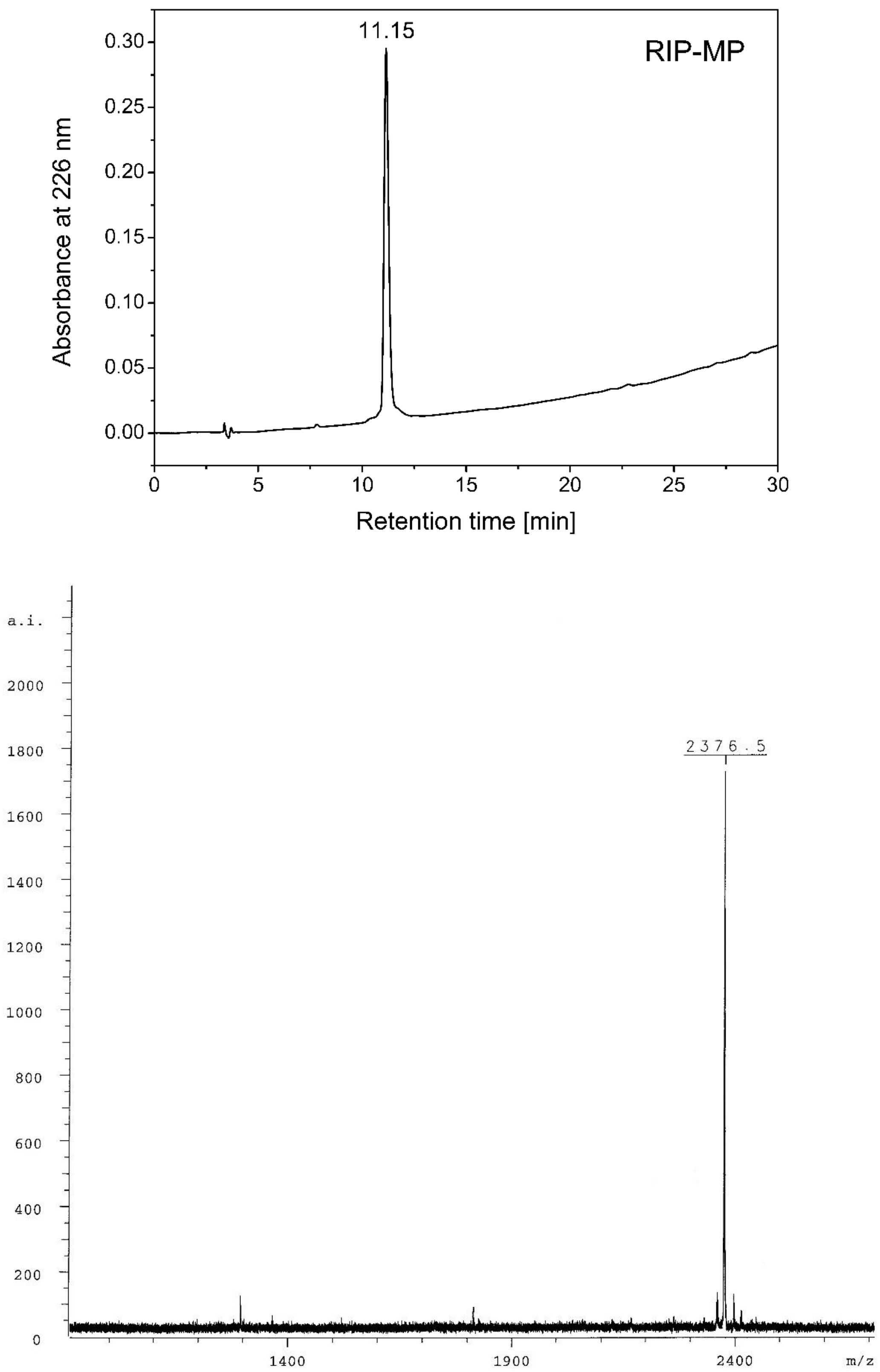

Figure S3. HPLC chromatogram (up) and MALDI-TOF mass spectrum (down) of RIP-MP.

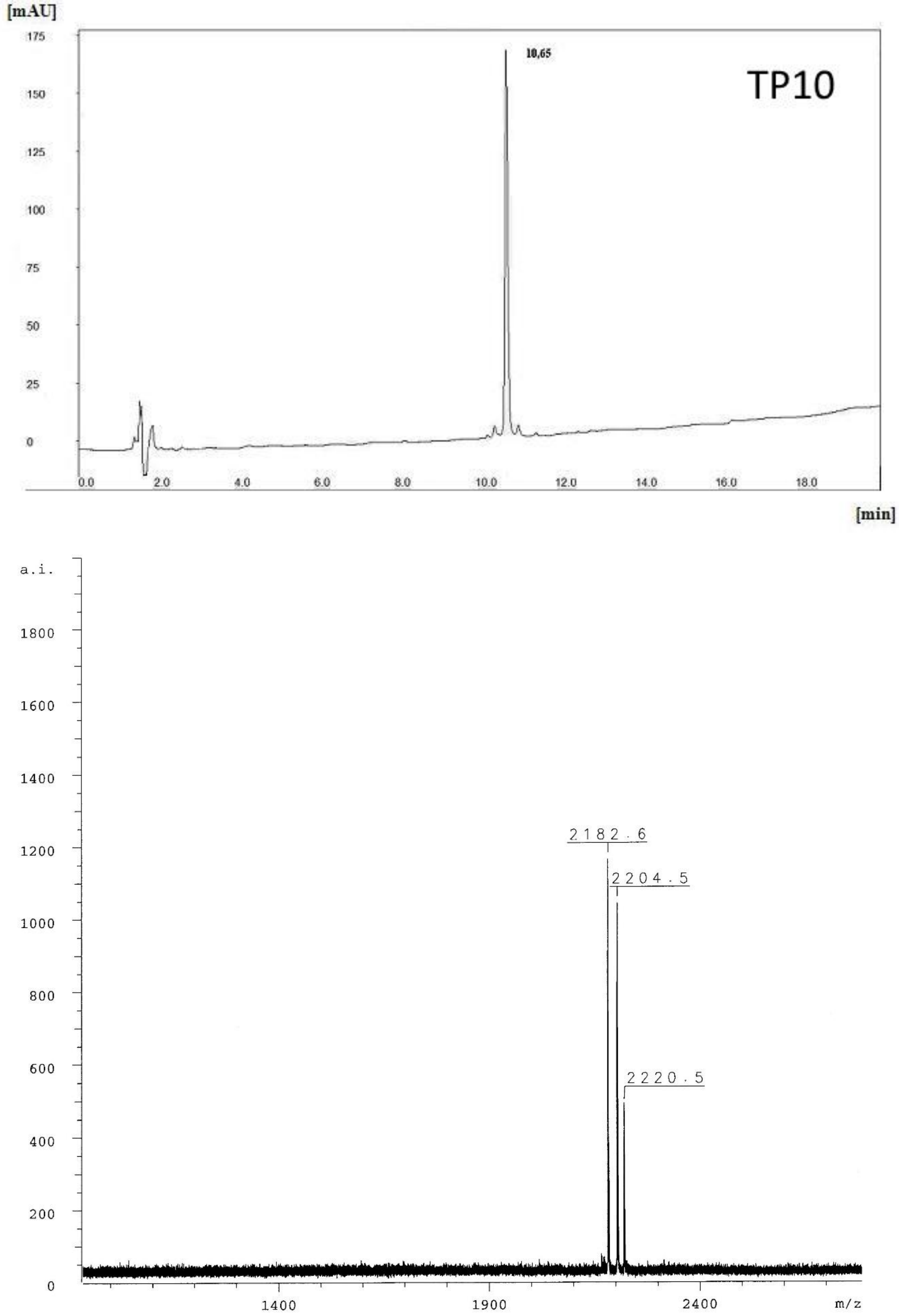

Figure S4. HPLC chromatogram (up) and MALDI-TOF mass spectrum (down) of TP10.

Table S1. Calculated and found molecular weight of synthesized peptides.

Peptide	Molecular weight	
	Calculated	Found
MP	1478.9	1479.1
retroMP	1478.9	1479.0
MP-retroMP	2940.9	2942.0
RIP	913.0	913.5
MP-RIP	2374.9	2375.0
RIP-MP	2374.9	2376.5
MP(4-14)	1138.5	1138.9
MP(4-14)-RIP	2034.5	2035.3
[Lys^2,Ile^4]RIP	881.0	881.3
MP(4-14)-[Lys^2,Ile^4]RIP	2002.5	2003.4
MP-[Lys^2,Ile^4]RIP	2342.9	2343.7
[Lys^2,Ile^4]RIP-MP	2342.9	2343.8
Galp	2809.4	2810.8
TP	2840.5	2040.8
TP10	2181.8	2182.6
TP10-RIP	3077.7	3078.8
TP10-[Lys^2,Ile^4]RIP	3045.8	3046.3
[desLys^7]TP10-[Lys^2,Ile^4]RIP	2917.6	2919.0
[Lys^7(BnzAc)]TP10	2339.8	2340.0
[Lys^7(NBnzAc)]TP10	2466.8	2467.0
[Lys^7(PBnzAc)]TP10	2417.4	2418.8
[Lys^7(ClBnzAc)]TP10	2450.4	2451.4
